# Gratitude and Religiosity in Psychiatric Inpatients with Depression

**DOI:** 10.1155/2024/7855874

**Published:** 2024-01-12

**Authors:** Silas R. S. Vandeventer, Michael Rufer, Micha Eglin, Harold G. Koenig, René Hefti

**Affiliations:** ^1^University of Zurich, Faculty of Medicine, Zurich, Switzerland; ^2^Department of Psychiatry, Psychotherapy and Psychosomatics, Psychiatric University Hospital Zurich, University of Zurich, Switzerland; ^3^Center for Psychiatry and Psychotherapy, Clinic Zugersee, Triaplus AG, Oberwil-Zug, Switzerland; ^4^Clinic SGM Langenthal, Clinic for Psychiatry and Psychotherapy, Langenthal, Switzerland; ^5^Research Institute for Spirituality and Health (RISH), Langenthal, Switzerland; ^6^Departments of Psychiatry and Medicine, Duke University Medical Center, Durham, USA; ^7^Department of Medicine, Division of Psychiatry, King Abdulaziz University, Jeddah, Saudi Arabia; ^8^University and University Hospital of Basel, Department for Psychosomatic Medicine, Basel, Switzerland

## Abstract

**Background:**

Gratitude and religiousness/spirituality are increasingly recognized resources that have potential influence on psychological states such as depression. However, only few studies have investigated this relationship in psychiatric patients.

**Objective:**

The present study examined gratitude in psychiatric inpatients with depression, exploring its relevance, course, and interaction with psychopathological and religious measures. Both general and religious gratitude will be evaluated.

**Methods:**

A total of 212 inpatients with depression completed a questionnaire both at the beginning and the end of treatment. Gratitude was measured with a general gratitude scale using the Gratitude Questionnaire and a religion-specific measure assessing gratitude to God as part of the Structure of Religiosity Test. The Beck Depression Inventory was used to evaluate depressive symptoms. General religiosity was assessed using the Centrality of Religiosity Scale.

**Results:**

Scores on the general and religious gratitude measures were in the upper range of these scales at baseline and demonstrated a significant increase during the hospital stay. Negative associations were found between general gratitude and depressive symptoms both on admission and at discharge (*r* = −0.505 and *r* = −0.478, respectively). General as well as religious gratitude was associated with the centrality of religiosity (*r* = 0.384 and *r* = 0.546, respectively). Religiosity accounted for approximately 10% of the variance in general gratitude on admission.

**Conclusions:**

Gratitude is highly prevalent in psychiatric patients with depression, and that may serve as a resource for these individuals. Both general and religious gratitude are associated with religiosity, which may also serve as a resource to these patients.

## 1. Introduction

Depression is a serious and growing threat to wellbeing, affecting more than 264 million people worldwide [1]. With its high risk for suicide, depression is the deadliest psychological disorder for all age groups, with suicide being the second leading cause of death in fifteen- to twenty-nine-year-olds [1, 2]. Since depression is a severe illness, looking for ways to improve treatment of depression becomes increasingly important, particularly since the burden of depression is globally rising [1].

Research on gratitude and religiousness/spirituality (R/S) has been increasing, including their relationship to depression. In a systematic review conducted by Braam and Koenig [3], these researchers found that about half of 152 prospective studies reported a significant inverse association between measures of R/S and the course of depression over time. Gratitude has also been increasingly recognized as a resource that influences depressive symptoms, stress, and subjective wellbeing in healthy patients [4, 5]. Yet only few studies on gratitude and depression have been conducted in clinical samples involving psychiatric patients. Previous studies have shown that patients with psychiatric disorders, such as depressions [6, 7], schizophrenia [6], and suicidal tendencies [8], benefit from gratitude. In addition, gratitude modifies pathophysiological pathways, such as reducing inflammation in heart failure patients, lowering blood pressure, and improving immune function [9]. By fostering prosocial behaviour and social support, gratitude enhances the quality of human relationships. This leads to better physical health and increased subjective wellbeing, which in turn promotes gratitude [4]. Gratitude can be understood in both “state” and “trait” gratitude. While state gratitude describes the extent to which gratitude is experienced in a given situation, trait gratitude refers to a consistent feeling of gratitude across situations.

Grateful feelings towards God or a higher being represent another aspect of gratitude. Rosmarin et al. [10] discussed the differences between gratitude in a religious and in a secular context. Gratitude in a secular context was conceptualized as interpersonal gratitude (i.e., being thankful to another human being). However, a person with a strong concept of God (or a higher power outside of the self) will explain positive life events as a benevolent act of God and therefore has additional reasons to feel grateful.

Several studies show that R/S is associated with increased levels of gratitude [7, 10]. Gratitude is at the core of many major religious traditions, and thanksgiving is a worldwide response to positive life events. Krause [11] reported that more frequent attendance at worship services leads to stronger God-mediated control beliefs. This, in turn, was associated with increases in gratitude over time.

Since most studies examining gratitude have been performed in nonclinical settings, the present study sought to investigate this construct in psychiatric patients with depression. The aims were to (1) evaluate how grateful depressed psychiatric patients are, (2) assess changes in gratitude over the course of treatment (from admission to discharge), (3) clarify how gratitude correlates with psychopathological and religious measures (depression, psychological burden, gratitude towards God, and centrality of religiosity), and (4) investigate the influence of religiosity on gratitude.

## 2. Methods

### 2.1. Recruitment and Data Collection

This is a retrospective study based on data collected at Clinic SGM Langenthal (Stiftung für ganzheitliche Medizin) in psychiatric inpatients suffering from depression (ICD 10 F32/F33), during a period of 3 years, from December 2004 until November 2007. To be included in the study, patients had to complete the questionnaires at admission and at discharge and give written consent to use their data for the present study. 212 patients out of 719 fulfilled these criteria.

### 2.2. Questionnaire

#### 2.2.1. Gratitude Scale

Gratitude is often measured with the Gratitude Questionnaire (GQ-6) [12]. For this study, the gratitude scale (GS), a modified version of the GQ-6, was used. That version includes three of the six GQ-6 items, as well as eight additional items to gain a broader view on gratitude [13]. All eleven items are rated on a Likert scale from 1 to 6 (strongly disagree to strongly agree), amounting to a total score from 11 to 66. The GS and GQ-6 have the following three questions in common: “I have so much in life to be thankful for,” “If I had to list everything that I felt grateful for, it would be a very long list,” and “I am grateful to a wide variety of people.” We compared the 3 items from the GQ-6 with the items from the GC to determine whether they measured the same “construct.” A bivariate correlation analysis showed that both on admission (*r* = 0.911^∗∗^) and at discharge (*r* = 0.917^∗∗^), the questionnaires highly correlate. With the available data, good internal consistency could be measured on admission (*α* = 0.92) and at discharge (*α* = 0.925).

#### 2.2.2. Gratitude towards God

Structure of Religiosity Test (SRT) [14] consists of two main domains, centrality (see below) and content (religious beliefs, experiences, and behaviours). SRT has an acceptable Cronbach's alpha between *α* = 0.85 and *α* = 0.93 [15]. Gratitude towards God (GTG) is a one-item subscale of the SRT. The GTG has a score range from 1 to 5.

#### 2.2.3. Centrality of Religiosity

Centrality of religiosity (CR) is another subscale of SRT and measures how central religiosity is in the personal construct of the patient [14, 15]. CR consists of five core dimensions, intellect, ideology, public practice, private practice, and experience with equal weighting, and reflects the religious and spiritual self-concept. This is categorized in three prototypes: the highly religious, the religious, and the nonreligious. Cronbach's alpha is 0.85 [15].

#### 2.2.4. Depression

Beck's Depression Inventory (BDI) is a self-report 21-item measure of depression. Each item is rated on a 4-point Likert scale from 0 to 3, resulting in a total score ranging from 0 to 63. The BDI is widely used and has good internal consistency of *α* = 0.88 [16].

### 2.3. Data Management and Statistics

Data were extracted from the Clinical Data Management System (CDMS) of the Clinic SGM Langenthal and transferred to IBM SPSS version 27. Descriptive analyses, correlations, and linear regression were used to address the different study aims.

### 2.4. Ethics

This study was granted an exemption from the requirement for ethics approval by the Cantonal Ethics Committee of Zürich (BASEC-Nr. Req-2018-00688). Patients at the Clinic SGM Langenthal gave written consent for the further use of their data for scientific reasons. All patient data have a distinct case-id, which was encrypted with a randomly generated project-id to anonymize the data (HFV article 26 paragraph 1). Only the director of the research department had access to the key that allocates case-id to project-id.

## 3. Results

### 3.1. Patient Characteristics

A total of 212 patients were identified for the study.  Table 1 shows the descriptive statistics of the patient population comparing means on admission and discharge. Age and sex were only assessed at admission. General gratitude was in the upper range of the scale with mean score of 46.5 (SD = 8.8) on admission and 50.7 (SD = 7.8) on discharge. A similar pattern was seen for religious gratitude with 3.6 (SD = 1.0) on admission and 3.9 (SD = 0.9) on discharge.

**Figure 1:**
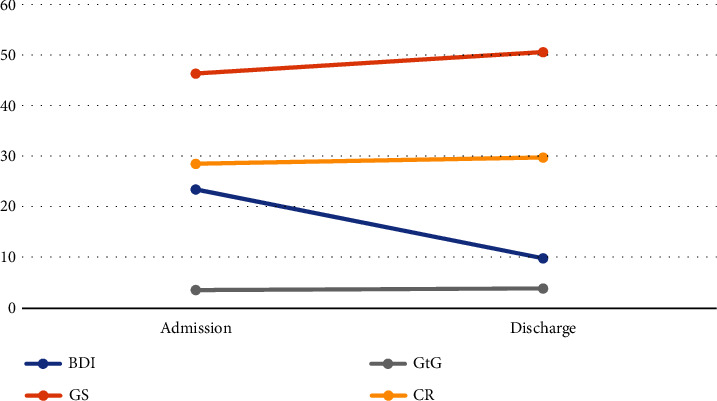
Means of main scales at admission and discharge. BDI: Beck Depression Index; GS: gratitude scale; GtG: gratitude towards God; CR: centrality of religiosity.

Table 1 and Figure 1 also show a significant increase of gratitude during inpatient treatment for general gratitude (8.3%; Δ = 4.24^∗∗^) as well as for religious gratitude (8.8%; Δ = 0.30^∗∗^). BDI decreased significantly by 57.9% (Δ = −13.19^∗∗^). There was also a small but significant increase in centrality of religion (4.4%; Δ = 1.08^∗∗^).

### 3.2. Association of Gratitude with Other Measures

The correlations between gratitude and psychopathological and religious measures are shown in Table 2. There is a significant negative correlation between general gratitude and BDI (*r* = −0.505^∗∗^/−0.478^∗∗^) on admission and discharge, documenting an inverse relationship between general gratitude and depressive symptoms. The same was found for religious gratitude (*r* = −0.274^∗∗^/−0.352^∗∗^) and depressive symptoms.

Table 2 also shows a positive association between general gratitude and centrality of religion (*r* = 0.384^∗∗^/0.366^∗∗^) as well as between religious gratitude and CR (*r* = 0.546^∗∗^/0.656^∗∗^) on admission and discharge. As expected, religiosity is more strongly associated with religious gratitude than with general gratitude. General gratitude is associated with religious gratitude on admission (*r* = 0.656^∗∗^) and discharge (*r* = 0.589^∗∗^). The higher correlation on admission might reflect a turning to religion during acute crisis.

Using a hierarchical regression model with general gratitude on admission as a dependent variable revealed a significant association with age (*β* = 0.248^∗∗^) with depression (*β* = −0.498^∗∗^) and religiosity (CR) (*β* = 0.326^∗∗^). The final model explained 41.7% of the variance of gratitude, with religiosity alone explaining 9.7% (Δ*R*^2^ = 0.097, *F* = 19.470, *p* < 0.01).

## 4. Discussion

The present study in depressed psychiatric inpatients found that (a) both general and religious gratitude scores were at the upper range of the scales, (b) there was a significant increase in general gratitude of 8.3% and religious gratitude of 8.8% during inpatient treatment, (c) negative associations were found both on admission and discharge between general gratitude and depression (*r* = −0.505^∗∗^/−0.478^∗∗^), and (d) religiosity, when controlling for BDI, sex, and age, explained 9.7% of the variance of general gratitude at admission. General and religious gratitude both were significantly associated with centrality of religion (GS: *r* = 0.384^∗∗^/0.366^∗∗^, GTG: *r* = 0.546^∗∗^/0.656^∗∗^). We now discuss these findings below.

Psychiatric inpatients in the present study showed the same levels of gratitude as study participants in nonclinical settings [8, 17]. This is rather surprising for a patient population suffering from depression. However, as seen in Results, religiosity and religious gratitude have a significant positive association with general gratitude. Therefore, the most obvious explanation is that the data was collected in a clinic with a high percentage of religious patients.

General and religious gratitude increased between admission and discharge, even though no direct interventions were undertaken to enhance gratitude. Several studies explored ways to increase gratitude in participants [6, 7, 9, 18]. The most common approach was gratitude journaling intervention, whereby participants had to write down three to five things they were grateful for on a daily basis [5]. Gratitude is based on a positive view of life. Therefore, a positive attitude towards life by the therapists might stimulate patients' gratitude without specific interventions.

Literature broadly agrees that gratitude is associated with increased subjective wellbeing [4, 18, 19] and with lower levels of depression [2, 7, 11, 17]. This study, by showing significant negative correlations between gratitude and depression, confirms these findings. Religiosity is commonly seen as having a positive influence on gratitude [7, 10, 11]. The results of the present study are in line with these findings. Wood et al. [17] showed that gratitude is associated with lower levels of depression over time, while depression did not influence gratitude over time.

Mosqueiro et al. [20] recently identified religious attendance as a main predictor of remission of depressive symptoms in severely depressed patients and observed that religious measures were inversely correlated to suicide risk scores. Furthermore, they discussed how the protective effects of religious attendance on depressive symptoms might be explained. Multiple factors, including religiously motivated positive mental attitudes (e.g., gratitude, forgiveness, or hope), are evaluated. The present study showed religiosity's predictive value for gratitude and thereby reinforces their argument.

### 4.1. Strengths and Limitations

Most studies investigating gratitude and its impact on psychological measures have been conducted in nonclinical settings. Therefore, a sample of 212 inpatients with clinical depression expands the scope of studies allowing to gain some further insight into the association of gratitude with clinical conditions. The interaction between general and religious gratitude specifies possible mechanisms.

Another strength of this study is having follow-up data, giving this retrospective analysis a longitudinal character, especially since only very few longitudinal studies on this subject can be found.

The main limitation of the study is its retrospective nature, limiting data analysis to the preexisting datasets. The gratitude scale (GS) used in this study is not a fully validated measure even though being based on a validated questionnaire (GQ-6).

Most of the studies on religious involvement and gratitude have been made within the Christian faith tradition. This is also true for the present study. It might be of interest to explore the interaction depression, gratitude, and religiosity in different faith traditions.

For further studies, a longitudinal prospective approach, investigating the correlations between gratitude at admission and the change (delta) of depressive symptoms, would be of interest.

## 5. Conclusions

The high level of gratitude in a psychiatric patient population and the significant inverse relationship with depressive symptoms reinforce the importance of considering gratitude as a resource in the clinical treatment of depression.

## Figures and Tables

**Table 1:** Demographics and means of main scales at admission and discharge.

	Admission			Discharge			Change	
	*N*	Mean	SD	*N*	Mean	SD	Δ	*p*
Age	212	40.76	12.70					
Sex (female)	212	0.75	0.43					
BDI	114	23.55	9.43	135	9.91	7.97	-13.19	<0.001
GS	212	46.48	8.79	212	50.72	7.75	4.24	<0.001
GtG	204	3.62	0.99	207	3.94	0.87	0.30	<0.001
CR	204	28.62	6.83	207	29.87	6.64	1.08	<0.001

BDI: Beck Depression Index; GS: gratitude scale; GtG: gratitude towards God; CR: centrality of religiosity; SD: standard deviation. Δ = discharge − admission.

**Table 2:** Pearson correlations with psychological, religious, and existential measures.

		GS ad	GS dis	GtG ad	GtG dis
BDI ad	*N*	-0.505^∗∗^	-0.398^∗∗^	-0.274^∗∗^	-0.203^∗^
		114	114	114	114
BDI dis	*N*	-0.258^∗∗^	-0.478^∗∗^	-0.152^∗^	-0.352^∗∗^
		135	135	135	132
CR ad	*N*	0.384^∗∗^	0.282^∗∗^	0.546^∗∗^	0.494^∗∗^
		204	204	204	201
CR dis	*N*	0.219^∗∗^	0.366^∗∗^	0.378^∗∗^	0.656^∗∗^
		207	207	201	207

^∗^ *p* ≤ 0.05 and ^∗∗^*p* ≤ 0.01 (2-tailed). Ad: admission; BDI: Beck Depression Index; CR: centrality of religiosity; Dis: discharge; GS: gratitude scale; GtG: gratitude towards God.

## Data Availability

The datasets used and analyzed during the current study are available from the corresponding author on reasonable request.
